# Towards use of MRI-guided ultrasound for treating cerebral vasospasm

**DOI:** 10.1186/s40349-016-0050-2

**Published:** 2016-02-29

**Authors:** Robert H. Bonow, John R. Silber, Dieter R. Enzmann, Norman J. Beauchamp, Richard G. Ellenbogen, Pierre D. Mourad

**Affiliations:** Department of Neurological Surgery, University of Washington, 325 9th Ave, Box 359924, Seattle, WA 98104 USA; Department of Radiology, University of California Los Angeles, 924 Westwood Blvd. Suite 805, Los Angeles, CA 90024 USA; Department of Radiology, University of Washington, RR-218 Health Science Building, 1959 NE Pacific St, Seattle, WA 98195 USA; Division of Engineering, University of Washington, 18115 Campus Way NE, Bothell, WA 98011 USA

**Keywords:** Subarachnoid hemorrhage, Cerebral vasospasm, Therapeutic ultrasound, Vasoconstriction, Brain

## Abstract

Cerebral vasospasm is a major cause of morbidity and mortality in patients with subarachnoid hemorrhage (SAH), causing delayed neurological deficits in as many as one third of cases. Existing therapy targets induction of cerebral vasodilation through use of various drugs and mechanical means, with a range of observed efficacy. Here, we perform a literature review supporting our hypothesis that transcranially delivered ultrasound may have the ability to induce therapeutic cerebral vasodilation and, thus, may one day be used therapeutically in the context of SAH. Prior studies demonstrate that ultrasound can induce vasodilation in both normal and vasoconstricted blood vessels in peripheral tissues, leading to reduced ischemia and cell damage. Among the proposed mechanisms is alteration of several nitric oxide (NO) pathways, where NO is a known vasodilator. While in vivo studies do not point to a specific physical mechanism, results of in vitro studies favor cavitation induction by ultrasound, where the associated shear stresses likely induce NO production. Two papers discussed the effects of ultrasound on the cerebral vasculature. One study applied clinical transcranial Doppler ultrasound to a rodent complete middle cerebral artery occlusion model and found reduced infarct size. A second involved the application of pulsed ultrasound in vitro to murine brain endothelial cells and showed production of a variety of vasodilatory chemicals, including by-products of arachidonic acid metabolism. In sum, nine reviewed studies demonstrated evidence of either cerebrovascular dilation or elaboration of vasodilatory compounds. Of particular interest, all of the reviewed studies used ultrasound capable of transcranial application: pulsed ultrasound, with carrier frequencies ranging between 0.5 and 2.0 MHz, and intensities not substantially above FDA-approved intensity values. We close by discussing potential specific treatment paradigms of SAH and other cerebral ischemic disorders based on MRI-guided transcranial ultrasound.

## Background

Aneurysmal subarachnoid hemorrhage is a devastating neurological condition with an estimated incidence of 7–9 per 100,000 person-years [1]. Roughly, one fifth of patients do not survive the initial bleed, and those who do remain at substantial risk for further morbidity and mortality. Overall death rates for subarachnoid hemorrhage range from 32 to 67 %, and 60 % of those who survive suffer from long-term disabilities, including cognitive difficulties, psychiatric illness, impaired activities of daily living, and inability to return to work [2].

In 20 to 40 % of patients, new ischemic neurological deficits that were not present on hospital admission become apparent in the days and weeks following the ictus [3]. Such delayed neurological deficits (DNDs) can be mild or severe, focal or non-focal, and reversible or permanent. Lethal infarctions occur in 7 % of cases [3].

## Review

### The pathophysiology of vasospasm: large vessels

Classically, these phenomena have been ascribed to spasm of large cerebral arteries leading to ischemia in downstream tissue, and a substantial body of evidence has been published in support of this conclusion [4–7]. For example, an early study by Fisher and colleagues demonstrated that DND occurred only in patients with severe vasospasm, and these deficits could frequently be attributed to a lesion in the brain region supplied by the spastic vessels [7].

Recently, however, there has been growing recognition that large-vessel spasm alone is not a sufficient explanation [8, 9]. For instance, angiographic narrowing of large-caliber vessels is evident in roughly two thirds of patients, but only one third actually exhibit symptoms [3]. Moreover, DNDs can occur in patients without large-vessel spasm [10, 11], and when vasospasm and a DND are present in the same patient, the clinical picture cannot always be attributed to a lesion in the region supplied by the affected vessel [8, 11]. Indeed, as many as one third of patients suffer infarctions in territories that could not be predicted by angiography [12]. Finally, there are conflicting reports on whether or not angiographic spasm and elevated blood velocity measured by transcranial Doppler (TCD) ultrasound—a measurement frequently used as a proxy for radiographic evidence of spasm—are ultimately associated with worse clinical outcome [13–15].

### The pathophysiology of vasospasm: cerebral microvasculature

In support of the idea that major-vessel vasospasm alone is not sufficient, there is a substantial body of evidence indicating that the cerebral microvasculature plays an important role. Post-mortem studies of subarachnoid hemorrhage (SAH) patients have demonstrated ischemic lesions that are much more frequently small and multifocal than they are large and territorial [16–18], and magnetic resonance imaging often shows multiple areas of restricted diffusion rather than a single, large infarction [18–20]. These patterns of injury are more consistent with widespread pathology in small vessels than they are with ischemia from large-vessel narrowing. Furthermore, while profound, large-vessel spasm can lead to significant drops in regional cerebral blood flow (rCBF), there is not always a clear link between perfusion and large-vessel diameter [11, 21]. There is, however, a strong and statistically significant correlation between small-vessel resistance and rCBF [11].

The mechanism by which small vessels contribute to ischemic injury is not well understood, but several hypotheses have been advanced. One that has received extensive attention relates to the brain’s ability to regulate its own perfusion. Though large-caliber vessels contribute proportionally more to vascular resistance in the brain than in other tissues [22], small cerebral arterioles remain the primary regulators of rCBF. In the healthy brain, decreased pressure from large artery constriction or systemic hypotension is compensated for by dilation of these arterioles, a process that maintains tissue perfusion within normal ranges. Small vessels also dilate in response to spikes in local neuronal firing, both to deliver the substrates required for metabolism and also to clear its by-products.

SAH appears to impair these homeostatic mechanisms. In one study, the severity of ischemic lesions was associated with labile blood pressure, a finding that could be explained by an inability of the brain to regulate blood flow appropriately in the face of drops in pressure [16]. Imaging studies and invasive tissue oximetry have provided more direct evidence that this autoregulatory response is absent, and there is some suggestion that the duration of impairment is related to subsequent DND [23, 24]. Mechanistic support for these clinical data are provided by animal work, which has shown that the normal physiologic dilation of both pial and intracerebral arterioles in response to increased neuronal activity is blunted temporarily after SAH [25, 26].

As discussed below, emerging evidence indicates that the pleiotropic signaling molecule nitric oxide (NO) and its downstream signaling pathways play a central role in the normal and pathological regulation of cerebral microvasculature.

### Current treatments for vasospasm

The substantial morbidity and mortality caused by SAH has long driven the search for effective treatments. While some interventions have shown promise, outcomes remain poor.

Hemodynamic therapy with induced hypertension and volume expansion was first used in the 1970s, and early series demonstrated that these therapies were effective in as many as 85 % of patients [27, 28]. In the decades since, they have become widely accepted interventions. By raising systemic blood pressure and decreasing blood viscosity, one can maintain flow in the distal vasculature despite the greater pressure drops across spastic proximal vessels, thereby preserving cerebral perfusion. It is not a completely benign treatment, however, as up to one third of patients suffer complications such as pulmonary edema, congestive heart failure, or myocardial infarction. Furthermore, high-quality evidence of improved patient outcomes is lacking [27, 29].

Whereas hemodynamic therapy works by circumventing the presumed source of ischemia, several treatments to address the spasm itself have been tried without great success. For example, clazosentan is an endothelin receptor A antagonist that showed initial promise for treating DND. In a phase 2 study, the drug reduced the incidence of moderate or severe radiographic large-vessel spasm from 66 to 23 % [29]. Despite this impressive effect, a follow-up phase 3 study showed no significant difference in the incidence of neurological deficits (21 vs. 25 %, *P* = 0.10) or poor long-term outcome (29 vs. 25 %, *P* = 0.10) [30]. Similarly, the vasodilatory calcium channel blocker nicardipine does not appear to improve long-term outcomes; in a randomized control trial, there was no significant effect of the drug on either infarct burden (129 vs. 148 cm^3^, *P* = 0.58) or the number of patients with poor long-term outcome (45 vs. 44 %, *P* = 0.46) [30]. Interventional approaches such as balloon angioplasty and intra-arterial vasodilator injection have also been employed with rates of radiographic improvement as high as 100 % in some series [31], but high-quality evidence of improved long-term neurological outcomes is wanting [29].

By contrast, the calcium channel blocker nimodipine has only modest effects on angiographic spasm, but it is the only agent that has been repeatedly demonstrated to have beneficial effects on outcome in multiple studies, including randomized control trials [32–34]. A meta-analysis concluded that the odds of a good outcome were 1.86 times higher in patients treated with nimodipine and the odds of infarction were reduced by a factor of 0.46 [35]. The mechanism of this effect is not well understood, but it may be related to effects on microvasculature or its ability to restore the local autoregulatory increase in blood flow that occurs with neuronal activity and which is lost in SAH [36].

Some authors have also found that HMG-CoA reductase inhibitors (statins) lower the risk of DND, with one meta-analysis concluding that treatment lowers the relative risk to 0.38 [37–39]. Though primarily used to reduce cholesterol synthesis and lower the risk of cardiovascular disease, this class of drugs is also known to modulate the inflammatory response, inhibit platelet activation, improve endothelial function, and upregulate expression of endothelial NO synthase (see below), which may underlie this benefit. While a recent randomized clinical trial failed to demonstrate evidence of improved outcomes as evaluated by the modified Rankin scale (odds ratio 0.97, *P* = 0.803), the duration of therapy was brief and detailed cognitive outcomes were not assessed [40, 41].

### Nitric oxide has multiple roles in brain physiology

NO may play an important role in mediating some of the beneficial effects of these treatments. NO is a pleiotropic signaling molecule that is produced by the enzyme nitric oxide synthase (NOS). Three isoforms of this protein are present in the brain and are expressed by distinct cell types: endothelial cells (eNOS), macrophages and microglia (inducible NOS, iNOS), and neurons and astrocytes (nNOS). As in other tissues, eNOS is constitutively active and produces a low basal level of NO that is important in vascular relaxation and in preventing platelet activation and thrombosis [42]. nNOS is constitutively expressed, and its activity helps to modulate neuronal signaling. The synthesis and release of NO by eNOS and nNOS occurs in response to normal physiological cues and together prevents vasospasm and vasoconstriction [43]. The inducible isoform, iNOS, is expressed primarily in microglia and astrocytes in response to inflammation and ischemic injury [44, 45]. The regulation of NOS synthesis, activity, and cellular localization is complex, involving transcriptional, post-transcriptional, and post-translational (e.g., phosphorylation) mechanisms [46].

In addition to its important intrinsic hemodynamic role, NO modulates intracellular signaling and neurotransmission through a variety of mechanisms [44]. The molecule has been found to protect against neuronal damage and death by blocking apoptosis, ameliorating excitotoxic injury, and inducing expression of antioxidants during inflammation [44]. Yet NO can also have toxic effects on neurons. During inflammation or excitotoxicity, NO produced by iNOS reacts with superoxide anions to form peroxynitrite-derived cytotoxic N_2_O· and OH· radicals, strong oxidizing agents that can damage cellular components and cause cell death [43, 47]. More directly, NO itself can cause cellular dysfunction through nitrosylation of serine residues on cellular proteins. It also perpetuates inflammation through induction of cyclooxygenase, the products of which include several pro-inflammatory mediators [44].

### Nitric oxide mediates vasospasm in SAH

Synthesis and release of NO into the vasculature occurs in response to normal physiological cues to induce cerebrovascular dilation [47]. However, vasospasm after SAH is associated with a 40 % reduction in NO elaboration by endothelial cells (via eNOS) and neurons (via nNOS) in the vessel adventitia [48, 49]. Moreover, oxidized heme products released from the initial hemorrhage scavenge free NO and elicit synthesis of endogenous eNOS inhibitors, such as asymmetric dimethylarginine [48]. In line with these findings, the normal vasodilatory response to exogenous NO precursors is blunted in animal models of SAH in a time course mirroring that of DND in humans [25, 26, 49].

Spreading ischemic injury exacerbates the situation by promoting the death of nNOS-expressing neurons. In addition, ischemia produced by vasospasm likely induces NO synthesis and release into the brain parenchyma (rather than in the cerebrovasculature) by microglia and astrocytes expressing iNOS [50]. As described above, high levels of parenchymal NO react readily with the superoxide anion that is produced in abundance in response to cerebral ischemia [51]. The resulting peroxynitrate reacts at physiological pH to produce free radicals, mediators of protein, and DNA damage that culminate in cell death.

Finally, there exist a few observations of the alteration of NOS isoform abundance and phosphorylation state in response to SAH [52, 53]. Of immediate relevance are the findings of Osuka et al. that active eNOS (i.e., phosphorylated at Ser1177) is transiently elevated by twofold in endothelial cells of the rat basilar artery 24 to 48 h after induction of SAH by puncture of the anterior cerebral artery [52]. Notably, iNOS content was detectable 6 h after SAH and was maximally expressed at 48 h, at which time, immunopositivity for nitrotyrosine (NT) was noted. Elevation of iNOS and NT in the vascular wall is indicative of nitrosative damage that may underlie the physiological and histological changes observed in arteries in vasospasm [52]. Therefore, suppression of iNOS production may reduce ischemic damage after SAH.

### Ultrasound—observations of peripheral vascular vasodilation and/or NO production and their potential relationship to cerebrovascular processes

Here, we briefly review the published work motivating our hypothesis that ultrasound (US) may reduce ischemic damage not through clot removal [54, 55], but through the complementary mechanism of vasodilation. Most studies have targeted the use of US for vasodilation of peripheral tissues, measured typically with US imaging with concurrent documentation of NO production or its direct correlates. These publications are summarized in Table 1. Many US protocols studied thus far in the context of NO production have used very low intensities (of order 0.2–1.0 W/cm^2^) pulsed with repetition frequencies on the order of 1 kHz (when not applied continually), across a range of US frequencies (0.02–3.0 MHz).

**Table 1:** Published literature regarding ultrasound-mediated vasodilation

Article	Model	US parameters	Results
Hightower and Intaglietta 2009 [60]	In vivo Hamster skin ischemia	• 2.5 MHz, continuous wave • 8 W/cm^2^ • 0.5 MPa • 20-min application to skin	• Microcirculation transiently decreased at 0.5 h but increased at 24 h • iNOS decreased at 2 h • eNOS increased at 24 h
Miyamoto et al. 2003 [56]	In vivo Canine coronary artery	• 27 kHz • 1.4 W/cm^2^ SPTA • Pulsed at 300 • 5-, 30-, 60-, and 90-min transcutaneous applications	• 9 % increase in the luminal area after 30 s of US, 19 % increase after 3 min • Return to baseline 90 min after cessation of US
Alexandrov et al. 2011 [65]	In vivo Transcranial after acute MCA occlusion	• 2 MHz • 0.128 or 0.010 W/cm^2^ • Transcranial application 1-h exposure after acute MCA occlusion	• Decrease in cerebral edema and infarction volume at 0.010 W/cm^2^ • No effect at 0.128 W/cm^2^
Suguta et al. 2000 [57]	In vivo Rabbit thigh adductor muscle	• 490 kHz, continuous wave • 0.25–1.7 W/cm^2^ SATA • 0.35–0.48 W/cm^2^ SPTA • Direct application	• NO production increased by 20 % • NO elaboration increases within 1 min of US application and decreases within 1 min of cessation • Effect partially ablated by NOS inhibitor L-NMMA
Suchkova et al. 2002 [71]	In vivo Ischemic rabbit thigh adductor	• 40 kHz • 0.25–0.75 W/cm^2^ • Direct application	• Tissue perfusion improves over 60-min US application • NOS activity increased; effect blocked by NOS inhibitor L-NAME
Altlad et al. 2004 [59]	In vitro HUVEC and BAEC cultures	• 27 kHz • 0.025–0.125 W/cm^2^, continuous wave • 0.25 W/cm^2^ pulsed 10 % duty cycle (10 Hz, 270 cycles per burst) SPTP • Direct exposure of variable duration, 10 s to 30 min	• Significant increase in NO elaboration and eNOS activity • Effect seen with 10-s application, increased up to 1-min application • Pulsed 10 % duty cycle yielded greater NO than continuous wave
Iida et al. 2006 [58]	In vivo Human brachial artery	• 29 kHz • 1.4 W/cm^2^ (0.12 W/cm^2^ ISATA) • Pulsed at 25 Hz, 30 % duty cycle • Transcutaneous application for 1, 2, 3, or 5 min	• Artery diameter increased after 2 min of US • Returned to baseline 21 min after cessation • Nonresponders were older
Hsu & Huang 2004 [72]	In vitro BAEC cultures	• 1 MHz 25 % pulsed • 0.5, 1.0, 1.6, and 2.0 W/cm^2^ ISATA (attenuated to 0.68 W/cm^2^) • 10 min, once daily for 6 days	• Increased extracellular matrix production in response to US • Culture medium NO increased approximately 50 % at 1.6 W/cm^2^ • Culture medium Ca^2+^ increased by approximately 20 % at 1.0, 1.6, and 2.0 W/cm^2^
Davis et al. 2015 [66]	In vitro Primary murine brain endothelial cells	• 1.05 MHz • 0.35, 0.55, 0.90, and 1.30 MPa • Pulsed at 50 Hz	• Increased production of various vasodilator molecules (e.g., adenosine, metabolites of arachidonic acid) in response to US • NO levels undetectable • eNOS phosphorylation unchanged

*HUVEC* human umbilical vein endothelial cells, *BAEC* bovine aortic endothelial cells, *MCA* middle cerebral artery, *US* ultrasound, *SATA* spatial average-temporal average, *SPTA* spatial peak-temporal average, *ISATA* spatial average-temporal average intensity

Broadly speaking, US applied peripherally has been shown to enhance blood flow within minutes of its application in both animal [56, 57] and human [58] models, mediated by the release of NO on time scales of seconds in vitro [59] to minutes in vivo [56–58]. In addition, US had longer lasting effects (measured up to 24 h [55]) on biological mechanisms that inhibit intraparenchymal NO production, a source of secondary injury, and enhance intravascular NO production, a source of therapeutic vasodilation.

Sugita et al. demonstrated a dose-dependent increase in NO elaboration by rabbit muscle tissue in response to US, with a maximal increase of 20 % when exposed to US with a power of 0.48 W/cm^2^ [57]. This effect was ablated by the application of L-NMMA, an NOS inhibitor, indicating that the increased NO production is at least in part due to NOS activity. Another study by Hightower and Intaglietta showed that US can increase blood flow in ischemic hamster skin in a manner accompanied by a 350 % increase in eNOS and a 60 % decrease iNOS [60]. However, these studies, which did not use an US contrast agent, provided no information on the phosphorylation state of eNOS or nNOS, and they do not explain the mechanisms by which US stimulates NOS production.

How might ultrasound affect NO production? NO production in the vasculature is sensitive to shear stress within the vessel walls, and ultrasound is able to generate shear stresses through two mechanisms: induction of bulk flow within fluids, also called acoustic streaming, and cavitation and with microstreaming around the resultant microbubbles [61–63]. We note that the intensity of ultrasound necessary to induce cavitation in brain tissue is 12.7 MPa, much higher than any of the protocols we have discussed [64], thereby favoring acoustic streaming as a means by which ultrasound may enhance NO production without causing damage to the parenchyma.

We know of only two publications relevant to the cerebral vasculature. In rats, Alexandrov and colleagues [65] reported significant reductions in the volume of edematous and ischemic brain with application of a commercially available TCD with a 2.0-MHz carrier frequency, 10-mW acoustic power, and 5-KHz pulse repetition frequency. They observed significant reductions in the volume of ischemic and edematous brain (75 vs. 22.5 mm^3^; 12.7 vs. 1.56 mm^3^, respectively) [65]. Given the unlikely occurrence of cavitation in the brain with these parameters [63, 64], we hypothesize this may have occurred through US-mediated enhancement of collateral flow in the volume of tissue surrounding the region of ischemic damage, due to local US-induced vasodilation of collateral blood vessels via stimulation of NO pathways as discussed, or production of enhanced flow via acoustic streaming. A second study by Davis et al. [66] using primarily cultured mouse brain endothelial cells suggests that that the reduction in infarct burden observed by Alexandrov and colleagues [65] may be at least in part related to elaboration of vasodilators such as adenosine and epoxyeicosatrienoic acids. This study did not find changes in NO levels or NOS activity, a discrepancy which may be due to differences in US parameters and the experimental model; for example, in vitro systems are more likely to experience US-induced cavitation, which may influence NOS activity and NO production.

### Discussion

A decrease in available NO contributes significantly to the initiation, persistence, and severity of cerebral vasoconstriction in SAH. There exist several studies demonstrating US-mediated induction of vasodilation via NOS production, but in peripheral tissue not in brain tissue. We hypothesize that US can improve blood flow in vasospasm by dilating spastic cerebral blood vessels and collaterals by elevating eNOS and nNOS activity via induction of shear stress in the vessel wall. We further hypothesize that US can suppress expression of iNOS, thereby limiting neurotoxicity.

If ultrasound can maintain or increase NOS production in an ischemic brain, we anticipate that extant MRI-guided therapeutic ultrasound systems could find use for the treatment of cerebral vasospasm via three focusing strategies. One would target ultrasound to the major cerebral arteries in vasospasm in a focal manner. A second focusing strategy would target generally large volumes of brain tissue containing secondary arteries in vasospasm by defocusing the nominally therapeutic ultrasound. The goal of these two strategies is to directly reverse vasospasm in these major and minor cerebral arteries. A third strategy would target collateral blood vessels that are available to perfuse brain tissue downstream from the spastic vasculature.

Fortunately, several studies support the idea that existing MRI-guided ultrasound systems could usefully treat ischemia due to vasospasm. For example, Sugita and colleagues used ultrasound consistent with existing MRI-guided ultrasound therapy systems (here, 490 KHz, approximately 0.2–0.5 W/cm^2^, useful for transcranial application) to demonstrate enhanced production of NO in vivo that scaled with the intensity of ultrasound [57]. Also, Hightower and Intaglietta also used an ultrasound protocol capable of transcranial delivery via extant MRI-guided ultrasound systems (2.95 MHz, 1 W/cm^2^, 5 KHz pulse repetition frequency) [60]. These are close to what existing MRI-guided therapeutic ultrasound systems can deploy: the Insightec system deploys ultrasound at 220 and 660 kHz. The Philips and SSI machines deploy ultrasound at 1 MHz. And, interestingly, the likely most efficacious ultrasound protocols should conform to FDA-approved guidelines for diagnostic ultrasound, given the low intensities necessary to create peripheral vasodilation, thereby reducing the regulatory barrier to clinical trials of MRI-guided therapeutic ultrasound to treat vasospasm in SAH.

If effective in manipulating CBF, this technology may also prove to have uses beyond DND after SAH. In ischemic stroke, for example, neuronal death begins minutes after the onset of ischemia, but tissue injury and cell loss progress over hours and even days in the penumbra [43], providing a crucial window for intervention. More broadly, perturbations in CBF feature prominently in neurological conditions not traditionally thought of as vascular in nature, such as migraine [67] and Alzheimer’s disease [68]. Should US prove effective in manipulating CBF and mitigating ischemia after SAH, perhaps it may find even broader indications.

Caution must be undertaken with any new therapy, however, and US is no different. Indeed, there is some evidence that US with a combination of high intensity and low frequency could be harmful [69]. Moreover, some have described vasoconstriction in response to lower intensities of US co-administered with acoustic contrast agents [70], which would obviously be counterproductive in patients with vasospasm. Clearly, significant research and development with an eye towards safety and efficacy are required before application of these ideas to patients.

## Conclusion

Delayed neurological deficits after subarachnoid hemorrhage remain a major cause of long-term morbidity and mortality, and the clinical tools available to treat the condition are fairly limited in efficacy. There exists a large body of literature implicating disordered NO signaling as a cause of this clinical problem. We hypothesize that application of US may induce cerebral vasodilation and restore blood flow to ischemic brain via increased elaboration of NO. In the future, MRI-guided therapeutic US systems may one day find their way through clinical trials and into regular clinical practice, most obviously as a treatment for cerebral vasospasm but also, possibly, for other cerebrovascular diseases.
